# Proteomic analysis reveals differential accumulation of small heat shock proteins and late embryogenesis abundant proteins between ABA-deficient mutant *vp5* seeds and wild-type *Vp5* seeds in maize

**DOI:** 10.3389/fpls.2014.00801

**Published:** 2015-01-20

**Authors:** Xiaolin Wu, Fangping Gong, Le Yang, Xiuli Hu, Fuju Tai, Wei Wang

**Affiliations:** State Key Laboratory of Wheat and Maize Crop Science, Collaborative Innovation Center of Henan Grain Crops, College of Life Science, Henan Agricultural UniversityZhengzhou, China

**Keywords:** abscisic acid (ABA), late embryogenesis abundant proteins (LEA proteins), maize ABA-deficient mutant *vp5*, mass spectrometry, protein abundance, proteome profiling, small heat shock proteins (sHSPs), 2-D electrophoresis (2-DE)

## Abstract

ABA is a major plant hormone that plays important roles during many phases of plant life cycle, including seed development, maturity and dormancy, and especially the acquisition of desiccation tolerance. Understanding of the molecular basis of ABA-mediated plant response to stress is of interest not only in basic research on plant adaptation but also in applied research on plant productivity. Maize mutant *viviparous*-5 (*vp5*), deficient in ABA biosynthesis in seeds, is a useful material for studying ABA-mediated response in maize. Due to carotenoid deficiency, *vp5* endosperm is white, compared to yellow *Vp5* endosperm. However, the background difference at proteome level between *vp5* and *Vp5* seeds is unclear. This study aimed to characterize proteome alterations of maize *vp5* seeds and to identify ABA-dependent proteins during seed maturation. We compared the embryo and endosperm proteomes of *vp5* and *Vp5* seeds by gel-based proteomics. Up to 46 protein spots, most in embryos, were found to be differentially accumulated between *vp5* and *Vp5*. The identified proteins included small heat shock proteins (sHSPs), late embryogenesis abundant (LEA) proteins, stress proteins, storage proteins and enzymes among others. However, EMB564, the most abundant LEA protein in maize embryo, accumulated in comparable levels between *vp5* and *Vp5* embryos, which contrasted to previously characterized, greatly lowered expression of *emb564* mRNA in *vp5* embryos. Moreover, LEA proteins and sHSPs displayed differential accumulations in *vp5* embryos: six out of eight identified LEA proteins decreased while nine sHSPs increased in abundance. Finally, we discussed the possible causes of global proteome alterations, especially the observed differential accumulation of identified LEA proteins and sHSPs in *vp5* embryos. The data derived from this study provides new insight into ABA-dependent proteins and ABA-mediated response during maize seed maturation.

## Introduction

Abscisic acid (ABA) is a major hormone that regulates a broad range of plant traits and is especially important for plant adaptation to environmental conditions. In seeds, ABA is thought to play a central role in many developmental stages, such as seed maturation and dormancy, the accumulation of nutritive reserves and the acquisition of desiccation tolerance (Quatrano, 1986). ABA-mediated plant response to stress has been extensively studied in different species ranging from Arabidopsis to crops, especially regarding ABA sensing, signaling, metabolism and transport (Umezawa et al., 2010). Knowledge about the complexity of ABA-mediated plant response to stress is still full of gaps, but the recent identification of ABA receptors (Ma et al., 2009; Santiago et al., 2009) and the key factors of the first step of ABA signal transduction (Park et al., 2009; Nishimura et al., 2010) in Arabidopsis provided an important insight into this mechanism.

Biosynthesis of ABA has been well characterized in Arabidopsis (Zeevaart and Creelman, 1988) and some data is available for other species, such as maize (Tan et al., 1997). Maize, *viviparous-5* (*vp5*) is deficient in ABA biosynthesis with the first step catalyzed by phytoene desaturase being blocked, which results in the precursor phytoene accumulation and carotenoid deficiency (Robichaud et al., 1980; Hable et al., 1998). Previous studies reported that ABA content in *vp5* embryos and endosperms was substantially reduced to 10 and 42% of the corresponding wild-type, respectively (Neill et al., 1986). The *vp5* seeds exhibit a visible phenotypic difference: the endosperm of mutant *vp5* seeds was white, while that of wild-type *Vp5* seeds was yellow. Therefore, *vp5* mutant is particularly useful not only for studies on the regulation of ABA-dependent maize genes, both in embryo and vegetative tissues, but also for studies of embryo development, seed germination and dormancy (Pla et al., 1989; Durantini et al., 2008).

Up to date, the expression of many individual gene/proteins has been studied using ABA-deficient mutant maize *vp5* and wild-type *Vp5* (Pla et al., 1989; Thomann et al., 1992). Williams and Tsang (1991) found *emb564* mRNA is expressed at low level in *vp5* embryos. The level of 3-hydroxy-3-methylglutaryl coenzyme A reductase activity, a rate-limiting enzyme of isoprenoid biosynthesis, is higher in *vp5* endosperm (Moore and Oishi, 1994). Recently, we compared root and leaf proteome differences between *vp5* and *Vp5* seedlings with 2-D gel electrophoresis (2-DE) combined mass spectrometry (MS/MS) and found that many proteins accumulation in roots or leaves are differentially regulated by drought and light in an ABA-dependent way (Hu et al., 2011, 2012). However, protein accumulation alterations caused by ABA-deficient mutation in *vp5* seeds are unclear at a proteome scale. Therefore, the characterization of seed proteome difference between *vp5* and *Vp5* is necessary for dissection of ABA-mediated maize response in the studies involved *vp5* mutants.

2-DE-based proteomics approach provides a powerful tool to analyze the expression levels of proteins, distinguish varieties and genotypes and even to identify single mutations with multiple effects (Lehesranta et al., 2005). This study aimed to characterize proteome alterations due to ABA-deficient mutation and further to identify ABA-dependent protein accumulation during seed maturation. We found significant proteome differences between *vp5* and *Vp5* seeds, where 46 differentially accumulated proteins were successfully identified. Most notably, six out of eight late embryogenesis abundant (LEA) proteins and nine small heat shock proteins (sHSPs) were found to differentially accumulate in ABA-deficient *vp5* embryos: six identified LEA proteins were repressed while nine sHSPs were induced.

## Materials and methods

### Plant materials

Maize (*Zea mays* L.) ABA-deficient mutant *vp5* mutant was provided by Maize Genetics Cooperation Stock Center (Urbana, IL). The mutant *vp5* was propagated in primarily W64 genetic backgrounds. The *vp5* mutant was maintained as a heterozygote. Heterozygous seeds (*Vp5/vp5*) were planted under natural conditions at the farm of Henan Agricultural University (Zhengzhou, China). The homozygous *vp5* kernels were identified on segregating ears by their lack of carotenoid pigments (Robichaud et al., 1980). The mutant *vp5* kernels appear white, while *Vp5* kernels are yellow. Mature *vp5* and *Vp5* seeds from the same ear were sampled (Presentation 1 in Supplementary Material) used in this study. Dry maize seeds were soaked in water for 2 h to soften starchy endosperm. For each biological replicate, the embryos and the endosperm of 20 maize seeds were manually separated and used for protein extraction, respectively.

### Protein isolation

Embryos or endosperms were powdered in liquid N_2_ and further ground in a buffer containing 0.25 M Tris-HCl (pH 7.5), 1% SDS, 14 mM DTT and a cocktail of protease inhibitors. This slurry was heated to 65°C for 5 min, vortexed, and heated at 95°C for 2 min, vortexed again, and then centrifuged at 12,000 g for 10 min to remove cellular debris. The supernatant was recovered and subjected to phenol extraction as described (Wu et al., 2014). The protein pellet was dissolved in 2-DE rehydration buffer containing 7 M urea, 2 M thiourea, 2% (w/v) CHAPS, 20 mM DTT, 0.5% (v/v) IPG buffer (pH 4–7 or 7–10, GE Healthcare). The protein content was determined by Bradford microassay (Bio-Rad) with BSA standards.

### SDS-PAGE and immunoblot

SDS-PAGE was performed in a Laemmli gel system (5% stacking gel and 12.5% resolving gel). After electrophoresis, proteins in gels were visualized with colloidal CBB R350 or electroblotted onto polyvinylidene difluoride membrane (Hybond-P, GE healthcare) in a transfer buffer (20% v/v methanol, 50 mM Tris, 40 mM glycine). For immunoblot analysis, protein blots were soaked in TBST buffer (50 mM Tris-HCl, pH 7.5, 0.15 M NaCl, 0.1% Tween-20) containing 5% low fat milk powder and gently shaken for 2 h at room temperature (RT). The blot was then incubated with anti-EMB564 polyclonal antibody (Wu et al., 2013a, 1: 5000 dilution) for 1 h. After washing with TBST, the blot was incubated in peroxidase-conjugated goat anti-rabbit IgG (1: 2000 dilution) at RT for 1 h. The blot was visualized with 0.08% 3,3′-diaminobenzidine tetrahydrochloride, 0.05% H_2_O_2_, 0.1 M Tris-HCl, pH 7.5.

### 2-DE, image and data analysis

Isoelectric focusing (IEF) was performed using 11-cm linear pH 4–7 IPG strips with the Ettan III system (GE Healthcare, USA). About 600 μg proteins were loaded into the strip by passive rehydration overnight at RT. The IEF voltage was set at 250 V for 1 h, 1000 V for 4 h, finally increasing to 8000 V for 4 h, and holding for 10 h (20°C). Focused strips were equilibrated in Buffer I (0.1 M Tris-HCl, pH 8.8, 2% SDS, 6 M urea, 30% glycerol, 0.1 M DTT) and then in Buffer II (same as Buffer I, but with 0.25 M iodoacetamide instead of DTT) for 15 min each. SDS-PAGE was run on a 13.5% gel with 0.1% SDS in the gel and the running buffer. The gels were stained with 0.1% CBB G-250 overnight and destained in 7% acetic acid until a clear background.

Protein gels were placed on a white plastic plate with transmission fluorescent lighting, and photographed using a DSLR camera (Nikon D7000) at an automatic mode. The digital images of the gels were analyzed by using PDQUEST 8.0 software (Bio-Rad). Protein spots were detected on scanned gels using the default spot detection setting. Gels of three biological replicates per genotype were analyzed. The spot intensities were normalized according to total intensity of valid spots to minimize possible errors due to differences in the amount of protein and staining intensity. Analysis of variance (ANOVA) was used to deal with protein spots quantification to identify individual protein spots with significantly different expression levels. Only those proteins with at least 1.5-fold quantitative variations in abundance were selected for mass spectrometry (MS) analysis.

### MS/MS

Protein spots of interest were manually excised from the gels and digested using trypsin. Proteins were reduced (10 mM DTT), alkylated (50 mM iodoacetic acid) and then digested with 10 mg/ml trypsin for 16 h at 37°C in 50 mM ammonium bicarbonate. The supernatants were vacuum-dried and dissolved in 10 μl 0.1% trifluoroacetic acid and 0.5 μl added onto a matrix consisting of 0.5 μl of 5 mg/ml 2, 5-dihydroxybenzoic acid in water: acetonitrile (2:1). The digested fragments were analyzed using a MALDI-TOF/TOF analyzer (ultraflex III, Bruker, Germany). MALDI-TOF/TOF spectra were acquired in the positive ion mode and automatically submitted to Mascot 2.2 (http://www.matrixscience.com, Matrix Science) for peptide mass finger printings against the NCBInr 20131226 database (35149712 sequences; 12374887350 residues, http://www.ncbi.nlm.nih.gov/). The taxonomy was Viridiplantae (green plants, 1669695 sequences). The search parameters were as follows: type of search: MALDI-TOF ion search; enzyme: trypsin; fixed modifications: carbamidomethyl (C); variable modifications: acetyl (protein N-terminal) and oxidation (M); mass values: monoisotopic; protein mass: unrestricted; peptide mass tolerance: ±50 ppm; fragment mass tolerance: ±0.2 Da; max missed cleavages: 1; instrument type: MALDI-TOF. Only significant scores defined by Mascot probability analysis greater than “identity” were considered for assigning protein identity. All of the positive protein identification scores were significant (*P* < 0.05, score > 49).

### Bioinformatics

To identify the sequences of all putative uncharacterized proteins, BLAST searches (http://www.expasy.org/tools/blast/) with these protein sequences were performed on the UniProt Knowledgebase (UniProtKB, http://www.uniprot.org/uniprot) to find their homologs. Functional categorization of the identified proteins was based on annotations in UniProtKB and previous studies on their homologs. Subcellular locations of the identified proteins were determined according to the annotation in UniProtKB or predicted at Plant-mPLoc server (http://www.csbio.sjtu.edu.cn/bioinf/plant-multi/). Theoretical Mr and isoelectric point of proteins were predicted at http://web.expasy.org/compute_pi/. ABA responsive element (ABRE) and dehydration responsive element (DRE) were analyzed using Plantcare (http://bioinformatics.psb.ugent.be/webtools/plantcare/html/) and PLACE (http://www.dna.affrc.go.jp/PLACE/signalscan.html).

## Results

Maize seed consists of an embryo (a miniature plant), an endosperm (a nutrition provider for seed germination) and a seed coat (protecting structure). To reveal maize seeds proteome alterations due to ABA-deficient mutation and to identify ABA-dependent proteins during seed maturation, embryos and endosperms of the mutant *vp5* and wild-type *Vp5* were used for comparative proteomic analysis. In order to improve protein resolution, two kinds of IPG strips with a pH range of 4–7 and 7–10 were used in 2-DE.

### Proteomic difference between *vp5* and *Vp5* embryos

**Figure 1:**
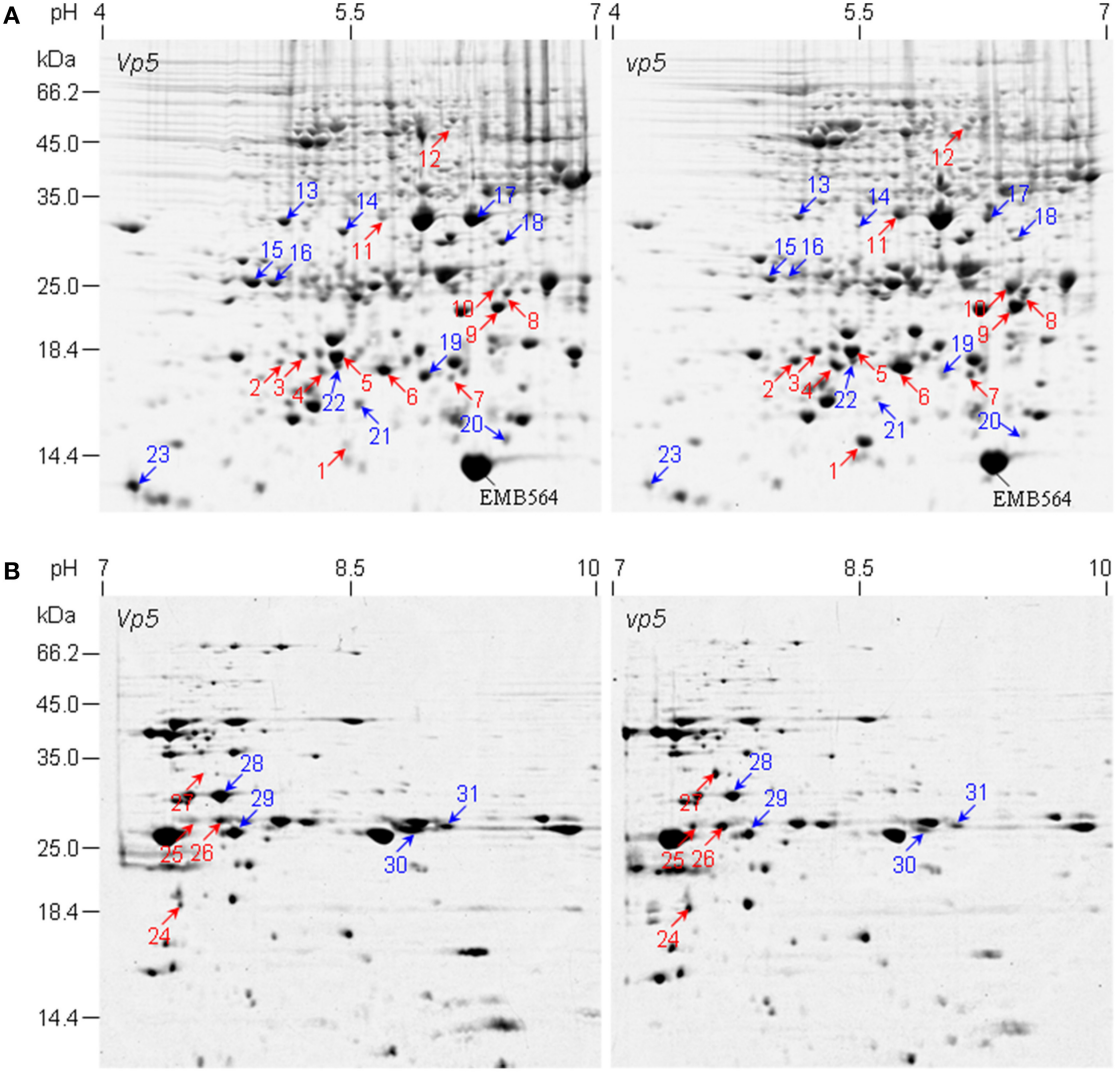
**2-DE comparison of embryo protein profiles between maize mutant *vp5* and wild type *Vp5***. **(A,B)** are representative 2-DE gels of pH 4–7 and pH 7–10, respectively. Differentially accumulated protein spots between *vp5* and *Vp5* embryos are indicated by arrows. *Red arrow*, increased abundance in *vp5*; *blue arrow*, increased abundance in *Vp5*. Embryo protein was extracted and resolved in 2-DE with IEF in the first dimension and 13.5% SDS-PAGE gel in the second dimension. Gels were stained with colloidal CBB G250. The identified proteins were listed in Tables 1, 2.

The embryo protein profiles between *vp5* and *Vp5* were compared by 2-DE. Approximately 780±10 and 130±5 CBB-stained protein spots were reproducibly detected using pH 4–7 and 7–10 IPG gels, respectively (Figure 1). PDQUEST analysis indicated that 96% of total protein spots were matched, unchanged in abundance between *vp5* and *Vp5* embryos across all the gels (Figure 1, Presentations 2, 3 in Supplementary Material). Spot-to-spot comparison revealed that 31 protein spots, i.e., 23 in pH 4–7 gels (Figure 1A and Presentation 2 in Supplementary Material) and 8 in pH 7–10 gel (Figure 1B and Presentation 3 in Supplementary Material), showed a minimum of a 1.5-fold difference in spot volume (Tables 1, 2). Those differentially accumulated embryo protein spots are mainly in the range of 10–35 kDa, with a consistent change in three biological replicates, except for three spots in two biological replicates (spot 5, 17, and 22) (Presentation 2 in Supplementary Material). Sixteen embryo protein spots (spots 1–12 and 24–27) accumulated in higher abundance in *vp5* compared to *Vp5* and 15 embryo protein spots (spots 13–23 and 28–31) accumulated in lower abundance in *vp5*. In particular, the abundance of spots 1, 10, and 27 were 13.71, 7.31, and 9.28 folds higher in *vp5* embryos than in *Vp5* embryos, respectively, whereas the abundance of spots 19 and 23 was 6.76 and 6.52 folds higher in *Vp5* than in vp5 embryos, respectively.

**Table 1:** **The differentially accumulated proteins identified by MS/MS between maize *vp5* and *Vp5* embryos**.

**Spot**	**Protein name**	**NCBI accession**	**pI Theor/exp**.	**MW (kDa) Theor/exp**.	**Mascot score**	**Coverage (%)**	**Matched peptide sequences**
1	Late embryogenesis abundant protein EMB564	gi|162463828	6.60/5.47	9.68/14.9	172	15	R.EQLGQQGYSEMGK.K
							R.EQLGQQGYSEMGK.K R.REQLGQQGYSEMGK.K
							R.REQLGQQGYSEMGK.K
2	17.8 kDa class II heat shock protein	gi|162459174	5.33/5.09	17.85/17.9	124	23	K.FVLPDNADVDK.V K.FVLPDNADVDKVAAVCR.D K.ELPGAYAFVVDMPGLGTGDIR.V
3	16.9 kDa class I heat shock protein 1	gi|226508268	5.82/5.21	17.28/18.2	248	22	R.VDWKETPEAHVFK.A
							R.SIVPSAASSGGGSETAAFANAR.V
4	17.4 kDa class I heat shock protein 3	gi|226530365	5.80/5.32	17.93/17.6	408	24	R.FRLPENAR.T
							R.VDWKETPEAHVFK.T K.VELEDGNVLQISGER.S
							K.EEVKVELEDGNVLQISGER.S
5	Heat shock protein 17.9	gi|1122317	5.55/5.41	17.78/18.1	506	32	R.FRLPENAK.T
							R.TSSETAAFAGAR.I
							R.IDWKETPEAHVFK.A R.IDWKETPEAHVFK.A
							K.VEVEDGNVLQISGER.N
							K.EEVKVEVEDGNVLQISGER.N
6	Heat shock protein 17.2	gi|162459222	5.54/5.68	17.15/17.6	474	38	K.VEVEDGNVLVISGQR.S
							R.SIVPSATSTNSETAAFASAR.I
							K.EEVKVEVEDGNVLVISGQR.S
							R.SNVFDPFSMDLWDPFDTMFR.S
							R.SNVFDPFSMDLWDPFDTMFR.S
7	17.0 kDa class II heat shock protein	gi|162458291	7.85/6.09	17.09/17.3	136	24	K.FVLPDNADVDKVAAVCR.D
							K.ELAGAYAFVVDMPGLSTGDIR.V
							K.ELAGAYAFVVDMPGLSTGDIR.V
8	22.0 kDa class IV heat shock protein	gi|226501206	6.72/6.41	25.22/24.9	792	33	R.EDLKIEVEDYSR.V R.ETPDAHEIVVDVPGMR.R
							R.ETPDAHEIVVDVPGMR.R
							R.LPENADLDSVGASLDNGVLTVR.F
							R.GGDEAAAAAASPLSGPGVGLLADPFR.I
							R.RGGDEAAAAAASPLSGPGVGLLADPFR.I
9	22.0 kDa class IV heat shock protein	gi|226509936	6.01/6.35	22.93/23.0	1063	43	K.LAPEQIKGPR.V R.ETPDAHEIVVDVPGMR.R
							R.ETPDAHEIVVDVPGMR.R
							R.GLDEAAVSDVGLLAADPFR.I
							RGLDEAAVSDVGLLAADPFR.I
							R.LPENADLDSVAASLDSGVLTVR.F
							R.ILEHVPFGFDRDDVAMVSMAR.V
							R.FRLPENADLPSVAASLDSGVLTVR.F
							R.FRLPENADLDSVAASLDSGVLTVR.F
10	1-Cys peroxiredoxin PER1	gi|162460575	6.31/6.31	25.06/25.0	350	21	K.VTFPILADPAR.D
							K.LSFLYPATTGR.N
							K.LLGISCDDVESHR.Q R.QLNMVDPDEKDAAGR.S
							R.QLNMVDPDEKDAAGR.S
11	Short-chain dehydrogenase/reductase SDR family protein	gi|226500748	5.78/5.67	33.15/34.9	525	27	R.DIGSETGAR.E
							R.ALSLQLADR.G
							K.EGATVAFTFVR.G
							R.GQEEKDAEETLR.A
							K.VEQFGSQVPMKR.A R.IDVVVNNAAEQYER.E
							R.EPMALPADLGYEANCR.E
12	UDP-glucose 6-dehydrogenase	gi|219885505	5.71/6.06	53.53/51.2	476	16	K.LAANAFLAQR.I
							K.AADLTYWESAAR.M
							K.DVYAHWVPEDR.I
							K.AQISIYDPQVTEDQIQR.D
							K.GINYQILSNPEFLAEGTAIEDLFKPDR.V
13	Late embryogenesis abundant protein D-34	gi|226530343	5.41/5.11	27.28/34.8	303	19	R.LNQERPRP.-
							R.RVVTESVGGQVVGK.M R.LQAAEQSVLGGTQK.G
							K.GGPAAVLQSAATVNAR.A
14	Late embryogenesis abundant protein D-34	gi|226530343	5.41/5.45	27.28/34.2	72	7	R.LNQERPRP.-
							R.VVTESVGGQVVGK.M
15	Late embryogenesis abundant protein D-34	gi|226493450	5.26/4.93	21.17/26.1	560	21	K.GAGGGVAEAVVAAADMNEGR.M
							K.IIEQGDQLGGALHVDQTDLPAGR.R
							R.DKIIEQGDQLGGALHVDQTDLPAGR.R
16	Late embryogenesis abundant protein D-34	gi|226501566	5.23/5.05	21.29/26.2	88	16	R.GGALHVDQTDLPAGR.R
							K.GAGGGVAEAVVAAADMNEGR.M
17	Short-chain dehydrogenase/reductase SDR family protein	gi|226500748	5.78/6.21	33.15/34.9	491	24	R.ALSLQLADR.G
							K.EGATVAFTFVR.G
							R.GQEEKDAEETLR.A
							K.VEQFGSQVPMKR.A R.IDVVVNNAAEQYER.E
							R.EPMALPADLGYEANCR.E
							R.EPMALPADLGYEANCR.E
18	Short-chain dehydrogenase/reductase SDR family protein	gi|226500748	5.78/6.38	33.15/32.5	492	27	R.DIGSETGAR.E
							R.ALSLQLADR.G
							K.EGATVAFTFVR.G
							R.GQEEKDAEETLR.A
							K.VEQFGSQVPMKR.A R.IDVVVNNAAEQYER.E
							R.EPMALPADLGYEANCR.E
							R.EPMALPADLGYEANCR.E
19	Hypothetical protein ZEAMMB73_326753	gi|413941572	5.93/5.95	11.50/17.4	511	54	R.AGPEEQTGR.G
							R.QGMAEPTAGGR.R
							R.TGFFDGTPLEGGK.I K.NAVIAESEPVDLPASAR.G
							R.VADAPYSSQQEGPHEEGTNK.N
20	Thioredoxin	gi|194694706	6.19/6.04	13.25/15.2	555	67	K.FTQVVFLK.V
							R.KDELLAQIEK.H
							R.AIAPLFVEHAK.K
							M.ASEQGVVIACHSK.A K.LVVIDFTAAWCGPCR.A
							K.VDVDEVKEVTAAYEVEAMPTFHFVK.N
							K.VDVDEVKEVTAAYEVEAMPTFHFVK.N
21	Glyoxalase family protein superfamily	gi|226532762	5.47/5.53	15.13/16.4	463	51	K.AASFYDAAFGYTVR.R
							R.ETDELSGAVQLPDSSAAGR.G
							R.GSVEVCFAYADVDAAYKR.A
							R.KWAELESGATTIAFTPLHQR.E
22	Late embryogenesis abundant protein Lea14-A	gi|226491145	5.64/5.41	16.14/17.7	458	38	R.TVASGTVPDPGSLAGDGATTR.L
							R.NPYSHAIPVCEVTYTLR.S
							K.LANIQKPEAELADVTVGHVGR.D
23	Uncharacterized protein	gi|223945515	5.73/4.23	22.86/13.7		27	R.YRGDPGPR.C
							K.TYPLTCSCFDR.V
							K.TYPLTCSCFDRVER.C R.CSDACKECVETEDSR.H
							R.DEERPWGECCDLAVCVK.T
24	17.4 kDa class I heat shock protein 3	gi|212276212	6.86/7.48	17.87/19.1	603	49	R.FRLPDNAK.A
							R.TSSETAAFAGAR.I
							R.IDWKETPEAHVFK.A K.VEVEDGNVLQISGER.N
							K.EEVKVEVEDGNVLQISGER.N
							R.GNAFDPFSLDLWDPFEGFFPFGSGGVR.S
25	Uncharacterized protein	gi|194693636	6.90/7.55	27.88/27.9	492	26	R.YGLSTEELR.A
							K.FWCTWQVDR.G
							K.IFDSLPAEEQR.L
							R.KIFDSLPAEEQR.L
							R.ADVEAPAEEHPGQADYWLR.H
							R.LNQDFLQCAVYDSDKADAR.L
26	Uncharacterized protein	gi|194693636	6.90/7.73	27.88/27.9	790	47	R.YGLSTEELR.A
							R.LIGVEYIVSR.K
							R.QVETHHYVSR.L
							K.FWCTWQVDR.G
							K.IFDSLPAEEQR.L
							R.KIFDSLPAEEQR.L K.SGLWTSPHVAGLLEK.A
							K.VLDMGAAAMQSLRPVK.Q
							R.ADVEAPAEEHPGQADYWLR.H
							R.LNQDFLQCAVYDSDKADAR.L
27	Uncharacterized protein	gi|413955864	6.60/7.63	38.12/33.3	57	12	R.GAAGGGGILESVQEGAR.S
							R.ETASTHDTDREQGQGLLGALGNVTGAIK.E
28	Uncharacterized protein	gi|194693636	6.90/7.73	27.88/30.4	923	47	R.YGLSTEELR.A
							R.LIGVEYIVSR.K
							R.QVETHHYVSR.L
							K.FWCTWQVDR.G
							K.IFDSLPAEEQR.L
							R.KIFDSLPAEEQR.L K.SGLWTSPHVAGLLEK.A
							K.VLDMGAAAMQSLRPVK.Q
							R.ADVEAPAEEHPGQADYWLR.H
							R.LNQDFLQCAVYDSDKADAR.L
29	Late embryogenesis abundant protein, group 3	gi|162463970	8.80/7.81	22.75/26.4	164	10	K.AAEAGQYAK.D
							K.SGGVIQQATEQVK.S K.DKSGGVIQQATEQVK.S
30	Globulin-1 S allele	gi|195658011	6.16/8.87	50.28/26.8	207	8	R.LLDMDVGLANIAR.G R.LLDMDVGLANIAR.G
							K.LLAFGADEEQQVDR.V
							R.HYEITGDECPHLR.L
31	Globulin-1 S allele	gi|195658011	6.16/9.09	50.28/27.2	353	14	R.GSMMAPSYNTR.A
							R.LLDMDVGLANIAR.G R.LLDMDVGLANIAR.G
							K.LLAFGADEEQQVDR.V R.HYEITGDECPHLR.L
							K.GQGYFEMACPHVSGGR.S
							K.GQGYFEMACPHVSGGR.S

**Table 2:** **The differentially accumulated proteins identified in maize *vp5* and *Vp5* embryos associated to putative functions**.

**Spot**	**Protein name**	**UniProtKB accession**	**Protein homology by Blast (Score/Identity)**	**Abundance change folds**	**Subcellular location**	**Molecular function**
**PROTEINS IN INCREASED ABUNDANCE IN *vp5* COMPARED TO *Vp5***						
**LEA proteins**						
1	Late embryogenesis abundant protein EMB564	P46517_MAIZE		13.1	Nucleus(Wu et al., 2013a)	Stress response
27	Uncharacterized protein	K7VM99_MAIZE	Group 3 LEA protein (1170/ 68.0%)	9.3	Cell wall^b^	Stress response
**HSPs**						
2	17.8 kDa class II heat shock protein	P24632_MAIZE		2.4	Cytoplasm^a^	Stress response; protein folding
3	16.9 kDa class I heat shock protein 1	B6T6N6_MAIZE		2.3	Nucleus^b^	Stress response; protein processing in ER
4	17.4 kDa class I heat shock protein 3	B6TLK8_MAIZE		2.4	Nucleus^b^	Stress response; protein folding; protein oligomerization
5	Heat shock protein 17.9	B6TDB5_MAIZE		2.5	Nucleus^b^	Stress response
6	Heat shock protein 17.2	Q43701_MAIZE		2.5	Nucleus^b^	Stress response; protein processing in ER
7	17.0 kDa class II heat shock protein	Q08275_MAIZE		2.1	Cytoplasm^a^	Stress response; protein processing in ER
8	22.0 kDa class IV heat shock protein	B6TG53_MAIZE		2.4	Plastid^b^	Stress response; protein processing in ER
9	22.0 kDa class IV heat shock protein	B6TXB5_MAIZE		1.5	Plastid^b^	Stress response; protein processing in ER
24	17.4 kDa class I heat shock protein 3	B4F976_MAIZE		2.8	Nucleus^b^	Stress response; protein processing in ER
**Oxidoreductase**						
10	1-Cys peroxiredoxin PER1	A2SZW8_MAIZE		7.3	Nucleus^a^	Phenylalanine metabolism; biosynthesis of other secondary metabolites
11	Short-chain dehydrogenase/reductase SDR family protein	B4FNZ9_MAIZE		3.6	Plastid^b^	Oxidation-reduction process
12	UDP-glucose 6-dehydrogenase	B7ZYX8_MAIZE		2.2	Plastid^b^	Cell wall pectin metabolic process
**Others**						
25	Uncharacterized protein	B4FFK9_MAIZE	Lipoprotein-like (1104/ 81.0%)	3.1	Plastid^b^	Unknown
26	Uncharacterized protein	B4FFK9_MAIZE	Lipoprotein-like (1104/ 81.0%)	2.1	Plastid^b^	Unknown
**PROTEINS IN REDUCED ABUNDANCE IN *vp5* COMPARED TO *Vp5***						
**LEA proteins**						
13	Late embryogenesis abundant protein D-34	B6UH67_MAIZE		2.2	Nucleus^b^	Stress response
14	Late embryogenesis abundant protein D-34	B6UH67_MAIZE		3.9	Nucleus^b^	Stress response
15	Late embryogenesis abundant protein D-34	B6SNS4_MAIZE		3.1	Nucleus^b^	Stress response
16	late embryogenesis abundant protein D-34	B6SN63_MAIZE		2.4	Nucleus^b^	Stress response
22	Late embryogenesis abundant protein Lea14-A	B6UH99_MAIZE		1.8	Plastid^b^ Nucleus^b^ Golgi apparatus^b^	Stress response
29	Late embryogenesis abundant protein, group 3	Q42376_MAIZE		2.2	Cell wall^b^	Stress response
**Enzymes**						
17	Short-chain dehydrogenase/reductase SDR family protein	B4FNZ9_MAIZE		1.7	Plastid^b^	Oxidation-reduction process
18	Short-chain dehydrogenase/reductase SDR family protein	B4FNZ9_MAIZE		2.0	Plastid^b^	Oxidation-reduction process
20	Thioredoxin	B4FH44_MAIZE		2.3	Cytoplasm^b^	Cell redox homeostasis; glycerol ether metabolic process; oxidation-reduction process
21	Glyoxalase family protein superfamily	B6SGF3_MAIZE		3.4	Cytoplasm^b^	Methylglyoxal metabolic process
23	Uncharacterized protein	C0HII8_MAIZE	Bowman-Birk serine protease inhibitor family protein (365/ 40.0%)	6.5	Extracellular region^a^	Negative regulation of endopeptidase activity
**Storage proteins**						
30	Globulin-1 S allele	B6UGJ0_MAIZE		3.6	Cell wall^a^	Seed maturation
31	Globulin-1 S allele	B6UGJ0_MAIZE		2.7	Cell wall^a^	Seed maturation
**Others**						
19	Hypothetical protein ZEAMMB73_326753	K7UCT3_MAIZE	Seed maturation protein (152/ 52.0%)	6.8	Nucleus^b^	Unknown
28	Uncharacterized protein	B4FFK9_MAIZE	Lipoprotein-like (1104/ 81.0%)	2.7	Plastid^b^	Unknown

a *Subcellular location of proteins was annotated in UniProtKB/Swiss-Prot (http://www.expasy.org/)*.

b *Subcellular location of proteins was predicted using the online Plant-mPLoc server (http://www.csbio.sjtu.edu.cn/bioinf/plant-multi/)*.

**Figure 2:**
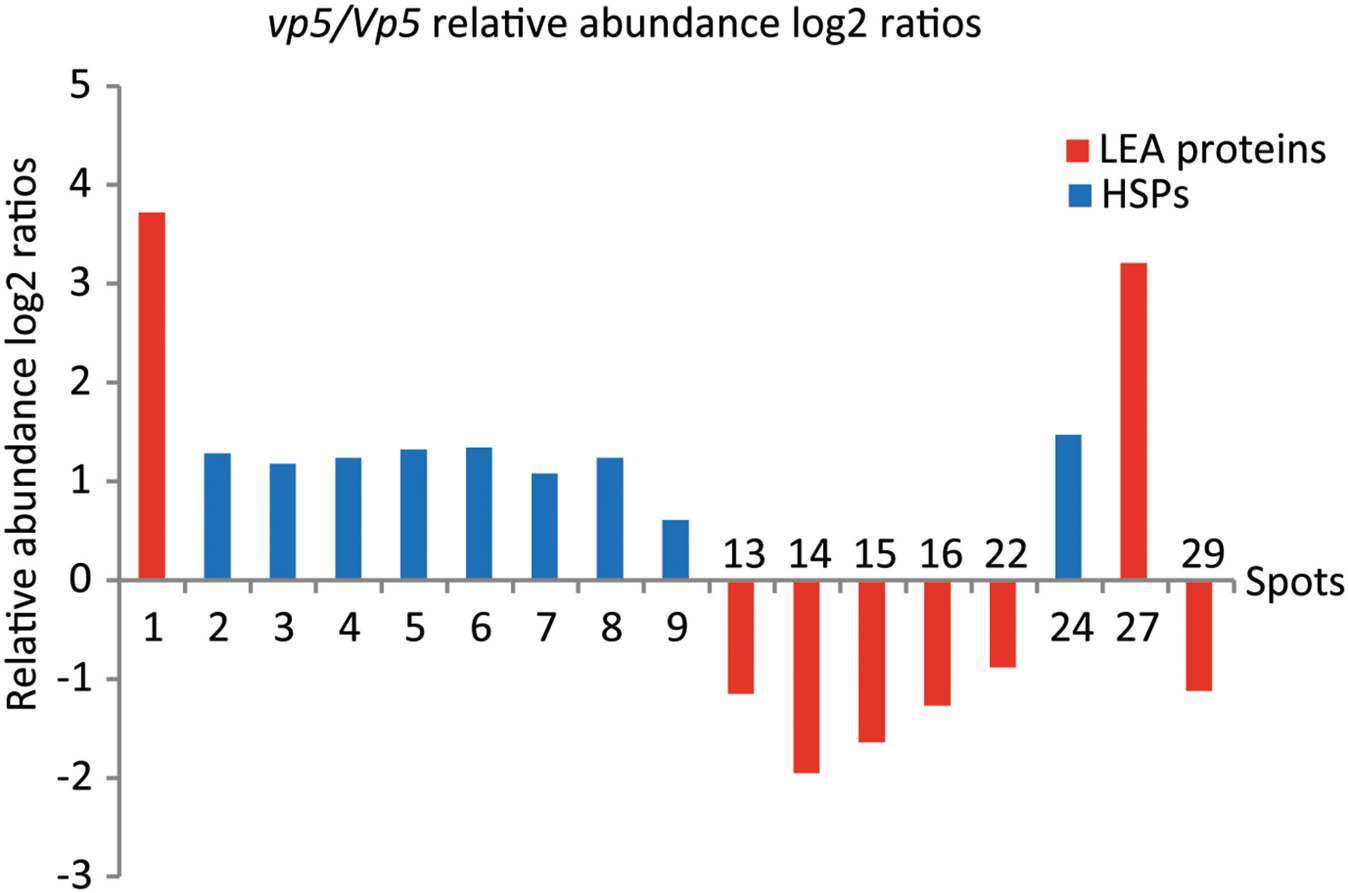
**The relative abundance log2 ratios of LEA proteins and sHSPs between *vp5* and *Vp5* embryos**. Log2 ratio > 1 represents abundance change more than two fold. Spot volumes were determined using the PDQuest software. Histograms full in red and blue represent LEA proteins and sHSPs, respectively.

The 31 embryo proteins were successfully identified by MS/MS analysis, representing 26 distinct proteins in NCBI or SWISS-PROT protein databases (Tables 1, 2). In several cases, proteins were identified as uncharacterized proteins. We searched their homologous proteins in other plant species by BLAST, such as spot 19 homologous to seed maturation protein of *Glycine latifolia* (identity of 40%) and spots 25, 26, and 28 homologous to lipoprotein-like of *Oryza sativa* (rice, identity of 81%), or were based on family and domain databases, such as spot 23 belonging to Bowman-Birk serine protease inhibitor family, and spot 27 belonging to group 3 LEA protein. These differentially accumulated protein spots were assigned to various LEA family proteins (spots 1, 13–16, 22, 27, and 29), HSP20 family proteins (spots 2–9 and 24), 1-Cys peroxiredoxin PER1 (spot 10), short-chain dehydrogenase/reductase SDR family protein (spot 11, 17, and 18), UDP-glucose 6-dehydrogenase (spot 12), seed maturation protein (spot 19), thioredoxin (spot 20), glyoxalase family protein (spot 21), Bowman-Birk serine protease inhibitor family protein (spot 23), lipoprotein-like (spot 25, 26, and 28), and globulin-1 S allele (spots 30 and 31). Nine sHSPs accumulated in higher abundance in *vp5* embryos, while six out of eight LEA proteins (except for spots 1 and 27) accumulated in higher abundance in *Vp5* embryos (Figure 2).

In the present study, two identified proteins belong to group 3 LEA protein (spot 27, K7VM99, spot 29 Q42376), but they exhibit opposite accumulation between *vp5* and *Vp5* embryos. In *vp5* embryos, Q42376, like other five identified LEA proteins, decreased in abundance, while K7VM99, like nine identified sHSPs, increased in abundance. This discrepancy may be explained by protein and gene sequence differences. K7VM99 and Q42376 share only a 35% identity in protein sequence alignment by BLAST.

**Figure 3:**
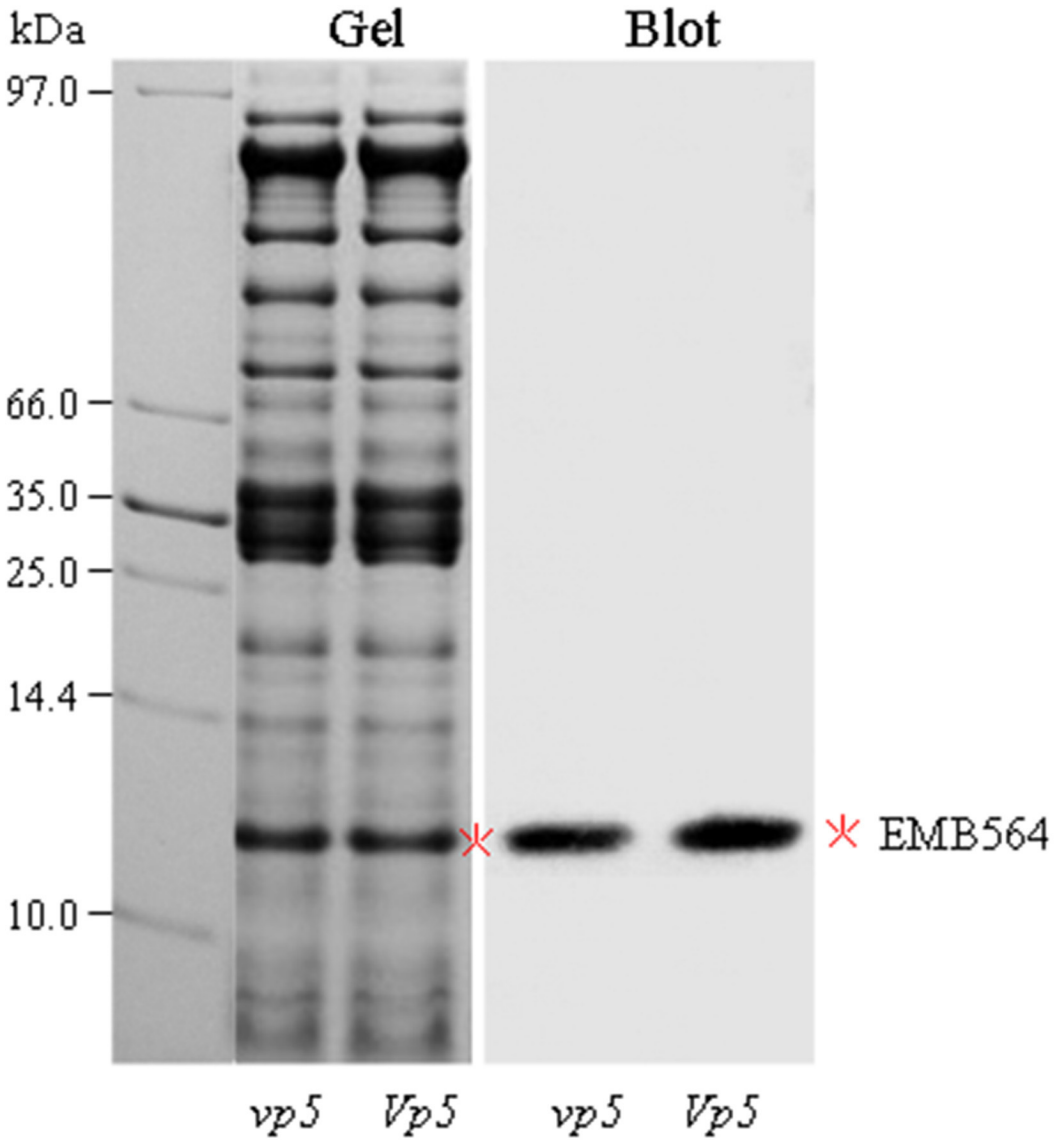
**Immunoblot detection of EMB564 in *vp5* and *Vp5* embryos**. Embryo protein (30 μg/lane) was resolved by 12.5% SDS-PAGE. Gel was stained with CBB. Blot was probed with an antibody specifically against EMB564 (1:5000 dilution). EMB564 was indicated by asterisk.

In addition, we found EMB564 existed in two isoforms: a weak spot 1 and a most predominant spot (indicated in Figure 1A). These two isoforms displayed a contrast accumulation in *vp5* compared to *Vp5*: the weak isoform (spot 1) increased greatly whereas the other decreased a little. However, the total abundance of EMB564 was comparable between *vp5* and *Vp5*. This result contrasted to previously characterized, greatly lowered expression of *emb564* mRNA in *vp5* embryos (Williams and Tsang, 1991). In order to further confirm the accumulation level of EMB564 in the two genotypes, we examined the abundance of EMB564 using immunoblot analysis (Figure 3). The specificity of the antibody has been recently characterized, and it specifically reacts with EMB564 (Wu et al., 2013a). Obviously, EMB564 existed in comparable levels between *vp5* and *Vp5* embryos, with a slightly reduced level in *vp5*.

### Proteomic difference between *vp5* and *Vp5* endosperms

In preliminary 2-DE experiments, none obviously differentially accumulated endosperms protein spots were observed to exist when pH 7–10 IPG gels were used (data not shown). Therefore, endosperm proteome analysis was performed only using pH 4–7 IPG gels.

**Figure 4:**
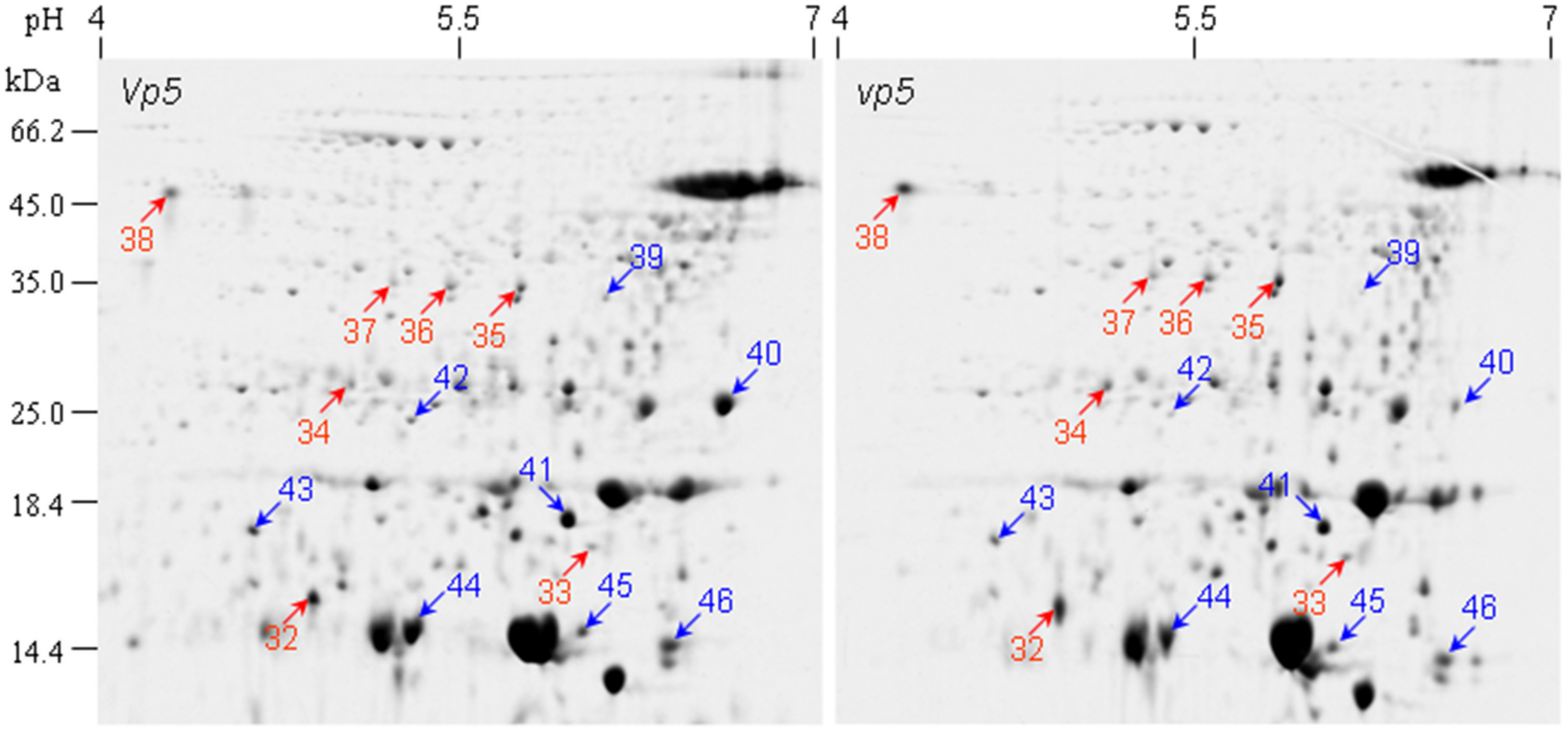
**2-DE comparison of endosperm protein profiles between maize *vp5* mutant and its wild type *Vp5***. Endosperm proteins were resolved by 2-DE as described in Figure 1.

The endosperm protein profiles between *vp5* and *Vp5* were compared by 2-DE. Approximately 380±10 CBB-stained protein spots were detected in endosperms, which was much less compared to embryos and most of proteins existed in low abundance (Figure 4). PDQUEST analysis indicated that 15 protein spots, especially spot 40, showed an obvious difference between *vp5* and *Vp5* endosperms, with a consistent change in three biological replicates (Figure 4, Presentation 4 in Supplementary Material). Seven spots (32–38) existed in higher abundance and eight spots (39–46) in lower abundance in *vp5*. These differentially accumulated protein spots were successfully identified by MS/MS (Tables 3, 4), representing 11 unique protein species.

**Table 3:** **The differentially accumulated proteins identified by MS/MS between maize *vp5* and *Vp5* endosperms**.

**Spot**	**Protein name**	**NCBI accession**	**pI Theor/exp**.	**MW (kDa) Theor/exp**.	**Mascot score**	**Coverage (%)**	**Matched peptide sequences**
32	Glyoxalase family protein superfamily	gi|226532762	5.47/4.90	15.13/15.7	783	51	K.AASFYDAAFGYTVR.R R.ETDELSGAVQLPDSSAAGR.G
							R.GSVEVCFAYADVDAAYKR.A K.WAELESGATTIAFTPLHQR.E
							R.KWAELESGATTIAFTPLHQR.E R.KWAELESGATTIAFTPLHQR.E
33	50 kD gamma zein	gi|162458022	6.83/6.06	35.40/17.2	459	23	K.NCHEFLR.Q
							R.CSYNYYSSSSNLK.N
							R.QQCSPLVMPFLQSR.L
							R.QQCSPLVMPFLQSR.L K.SQQQQCHCQEQQQTTR.C
							R.LIQPSSCQVLQQQCCHDLR.Q
34	Uncharacterized protein	gi|223944797	6.05/5.06	26.45/27.1	594	27	K.VSFGENFSPAR.A
							K.VKEGVEVAQLVEK.V
							R.SPAAEALGPTHLLHTR.Y R.LRSPAAEALGPTHLLHTR.Y
							K.GYQFGMVAVFDSVEELDAVEGDGKVEEAK.A
35	Lactoylglutathione lyase	gi|195639070	6.62/5.76	35.31/34.5	352	24	R.MLHAVYR.V
							K.YYTECFGMK.L
							K.IASFVDPDGWK.V
							R.ADTPEPLCQVMLR.V
							R.ADTPEPLCQVMLR.V
							K.VVLVDNTDFLKELH.-
							K.GGSTVIAFAQDPDGYMFELIQR.A
36	Lactoylglutathione lyase	gi|195639070	6.62/5.47	35.31/34.6	438	24	R.MLHAVYR.V
							K.YYTECFGMK.L
							K.IASFVDPDGWK.V
							K.VVLVDNTDFLK.E
							R.ADTPEPLCQVMLR.V
							K.VVLVDNTDFLKELH.- K.GGSTVIAFAQDPDGYMFELIQR.A
37	Lactoylglutathione lyase	gi|195639070	6.62/5.23	35.31/35.0	391	24	R.MLHAVYR.V
							K.YYTECFGMK.L
							K.IASFVDPDGWK.V
							K.VVLVDNTDFLK.E
							R.ADTPEPLCQVMLR.V
							K.VVLVDNTDFLKELH.-
							K.GGSTVIAFAQDPDGYMFELIQR.A
38	Maize protease inhibitor	gi|75994143	5.63/4.32	7.49/48.8	462	75	R.IFVDIVAQTPHIG.-
							K.TSWPEVVGLSVEDAK.K K.TSWPEVVGLSVEDAKK.V
							K.TSWPEVVGLSVEDAKK.V K.DKPDADIVVLPVGSVVTADYRPNR.V
39	Short-chain dehydrogenase/reductase SDR family protein	gi|226500748	5.78/6.12	33.15/33.7	575	29	R.ALSLQLADR.G
							K.EGATVAFTFVR.G
							R.ALRDIGSETGAR.E
							R.GQEEKDAEETLR.A
							K.VEQFGSQVPMKR.A
							R.IDVVVNNAAEQYER.E R.VASAYGGRIDVVVNNAAEQYER.E
							R.IDVVVNNAAEQYERESIGDVTEADLER.V
40	1-Cys peroxiredoxin PER1	gi|162460575	6.31/6.62	25.06/25.5	941	48	K.VTFPILADPAR.D
							K.LSFLYPATTGR.N
							K.LLGISCDDVESHR.Q
							R.QLNMVDPDEKDAAGR.S, K.MFPQGFETADLPSKK.G
							M.PGLTIGDTVPNLELDSTHGK.I
							K.VATPANWKPGECAVIAPGVSDEEAR.K
							K.VATPANWKPGECAVIAPGVSDEEARK.M
41	Superoxide dismutase [Cu-Zn] 4AP	gi|13431904	5.65/5.96	15.22/17.9	315	21	K.AVAVLGSSEGVK.G
							R.AVVVHADPDDLGK.G R.AVVVHADPDDLGKGGHELSK.T
42	Globulin-1 S allele	gi|195658011	6.16/5.30	50.28/24.4	448	16	R.VAELEAAPR.T
							K.EGEGVIVLLR.G
							K.GEITTASEEQIR.E
							R.FTHELLEDAVGNYR.V
							K.QSKGEITTASEEQIR.E R.EGDVMVIPAGAVVYSANTHQSEWFR.V
43	Superoxide dismutase [Cu-Zn]	gi|167860184	5.45/4.66	21.00/17.6	515	25	K.GGHELSLSTGNAGGR.L K.GASEVEGVVTLTQDDDGPTTVNVR.I
							R.AFVVHELEDDLGKGGHELSLSTGNAGGR.L
44	Trypsin/factor XIIA inhibitor	gi|157830250	8.07/5.30	16.30/14.8	479	46	R.LPWPELKR.R
							R.LEDLPGCPR.E
							R.ELADIPAYCR.C -.SAGTSCVPGWAIPHNPLPSCR.W
							R.CTALSILMDGAIPPGPDAQLEGR.L
							R.CTALSILMDGAIPPGPDAQLEGR.L
45	Glycine-rich RNA-binding protein 2	gi|195612516	6.10/6.02	15.48/14.9	652	54	M.AASDVEYR.C,
							R.NITVNEAQSR.G,
							K.IILDRETQR.S,
							R.GGGYGNSDGNWRN.-
							R.GFGFVTFSTEEAMR.N
							R.GFGFVTFSTEEAMR.N
							R.DGGGGYGGGGGYGGGGGYGGGGGGYGGGNR.G
46	Trypsin/factor XIIA inhibitor	gi|157830250	8.07/6.39	16.30/14.5	455	46	R.LPWPELKR.R
							R.LEDLPGCPR.E
							R.ELADIPAYCR.C -.SAGTSCVPGWAIPHNPLPSCR.W
							R.CTALSILMDGAIPPGPDAQLEGR.L

**Table 4:** **The differentially accumulated proteins identified in maize *vp5* and *Vp5* endosperms associated to putative functions**.

**Spot**	**Protein name**	**UniProtKB accession**	**Protein homology by Blast (Score/Identity)**	**Abundance change folds**	**Subcellular location**	**Molecular function**
**PROTEINS IN INCREASED ABUNDANCE IN *vp5* COMPARED TO *Vp5***						
**Enzymes**						
32	Glyoxalase family protein superfamily	B6SGF3_MAIZE		1.5	Cytoplasm^b^	Methylglyoxal metabolic process
35	Lactoylglutathione lyase	B6TPH0_MAIZE		1.6	Cytoplasm^b^	Metabolic process
36	Lactoylglutathione lyase	B6TPH0_MAIZE		1.5	Cytoplasm^b^	Metabolic process
37	Lactoylglutathione lyase	B6TPH0_MAIZE		1.7	Cytoplasm^b^	Metabolic process
38	Maize protease inhibitor	Q2XX01_MAIZE		1.9	Plastid^b^ Nucleus^b^	Proteolysis; response to wounding
**Storage proteins**						
33	50 kD gamma zein	Q946W1_MAIZE		2.1	Nucleus^b^	Seed maturation
**Stress-responsive protein**						
34	Uncharacterized protein	C0HHH9_MAIZE	Stress responsive alpha-beta barrel domain protein (371/42.0%)	1.5	Plastid^b^	Stress response
**PROTEINS IN REDUCED ABUNDANCE IN *vp5* COMPARED TO *Vp5***						
**Enzymes**						
39	Short-chain dehydrogenase/reductase SDR family protein	B4FNZ9_MAIZE		2.3	Plastid^b^	Oxidation-reduction process
40	1-Cys peroxiredoxin PER1	A2SZW8_MAIZE		5.2	Nucleus^a^	Phenylalanine metabolism; biosynthesis of other secondary metabolites
41	Superoxide dismutase [Cu-Zn] 4AP	P23346_MAIZE		2.0	Cytoplasm^a^	Superoxide metabolic process
43	Superoxide dismutase [Cu-Zn]	B1PEY4_MAIZE		1.7	Plastid^b^	Superoxide metabolic process
44	Trypsin/factor XIIA inhibitor	P01088_MAIZE		2.0	Extracellular region^a^	Negative regulation of endopeptidase activity
46	Trypsin/factor XIIA inhibitor	P01088_MAIZE		1.5	Extracellular region^a^	Negative regulation of endopeptidase activity
**Storage proteins**						
42	Globulin-1 S allele	B6UGJ0_MAIZE		2.5	Cell wall^a^	Seed maturation
**Other**						
45	Glycine-rich RNA-binding protein 2	B6STA5_MAIZE		1.7	Plastid^b^ Nucleus^b^	DNA duplex unwinding; RNA secondary structure unwinding; mRNA export from nucleus; Stress response

a *Subcellular location of proteins was annotated in UniProtKB/Swiss-Prot (http://www.expasy.org/)*.

b *Subcellular location of proteins was predicted using the online Plant-mPLoc server (http://www.csbio.sjtu.edu.cn/bioinf/plant-multi/)*.

## Discussion

### Global proteome alterations in embryo and endosperm of maize *vp5* and *Vp5*

Maize viviparous mutants, germinating directly on the ear (McCarty et al., 1991), are widely used for studying maize seed maturation, dormancy, and germination. Various viviparous mutants have been identified, such as *vp1*、*vp2*、*vp5*、*vp7*、*vp8*、 and *vp9*. Among them, *vp5* mutant is deficient in ABA biosynthesis with the first step catalyzed by phytoene desaturase being blocked, resulting in the precursor phytoene accumulation and carotenoid deficiency (Robichaud et al., 1980). However, the background difference at proteome level between *vp5* and *Vp5* seeds is still unclear.

In the present study, comparative proteomics was used to determine the variation of protein expression at the proteome level between maize mutant *vp5* and its wild type *Vp5*. There are great differences in the structure, composition and function between embryo and endosperm of maize seeds. Compared to endosperm, embryo is more active in nucleic acid, protein, and lipid metabolism. In total, 46 seed protein spots were found to be exhibited a differential change in abundance between these two genotypes, 31 spots in embryo and 15 spots in endosperm. Obviously, proteome alterations caused by ABA-deficient mutation are more significant in embryos than in endosperms in *vp5* seeds. This may be explained by two possible causes: Firstly, ABA deficiency mainly takes place in *vp5* embryos. Due to deficient in ABA biosynthesis, maize *vp5* embryos contains a low ABA content (about 10% of *Vp5*) throughout seed maturation, whereas 42% ABA in *vp5* endosperms (Neill et al., 1986). ABA in endosperm can be from maternal organs (Ober and Setter, 1992). Secondly, maize endosperms act mainly as starch storage tissue and contain fewer proteins in low amounts in mature seeds. Although ABA-deficient mutation affects the phenotype of *vp5* endosperm (carotenoid deficiency), proteins or enzymes involved in the related pathways were not detected, probably due to their low abundance and low sensitivity of CBB staining.

In the present study, four proteins, i.e., 1-Cys peroxiredoxin PER1 (spot 10 in embryo, spot 40 in endosperm), globulin-1 s allele (spot 30 and 31 in embryo, spot 42 in endosperm), glyoxalase family protein (spots 21 in embryo and 32 in endosperm) and short-chain dehydrogenase/reductase SDR family protein (spot 11, 17, and 18 in embryo, spot 39 in endosperm) were the same as identified embryo proteins. Among them, globulin-1 s allele (spot 30, 31, and 42) and short-chain dehydrogenase/reductase SDR family protein (spot 17, 18, and 39) decreased both in *vp5* embryo and endosperm compared to *Vp5*.

It is worth to note that EMB564, the most abundant LEA protein in maize embryos, was found to exist in comparable levels between *vp5* and *Vp5* embryos. EMB564 consists of two isoforms with obvious differences in apparent and theoretical values of sizes and isoelectric points, indicating a post-translational modification of this protein (e.g., phosphorylation, Amara et al., 2012). Williams and Tsang (1991) first cloned and characterized an embryo-specific recombinant, termed *emb564* [recently renamed as *embryo specific protein 1* (*esp1*) in NCBI database]. The *emb564* mRNA is expressed at low level in ABA-deficient (e.g., *vp5*) but not in ABA-insensitive (e.g., *vp1*) embryos during embryogenesis, and exogenous ABA has little effect on the accumulation of *emb564* mRNA in more mature embryos (Williams and Tsang, 1991). Therefore, there is a discrepancy between EMB564 protein and mRNA levels.

The transcript/protein discordance has been well documented in mammals, yeasts and plants (mainly Arabidopsis and maize). It is largely of biological origin (“true discordance”) and of post-transcriptional regulation (Vélez-Bermúdez and Schmidt, 2014). For example, in *Arabidopsis* roots, a lack of correlation between down-regulated transcripts and the amount of their corresponding proteins was observed in response to phosphate deficiency, whereas for induced genes changes in the levels of mRNAs and proteins were reasonably well correlated (Lan et al., 2012). In the ABA-deficient *vp5* embryos, EMB564 can accumulate to a similar level as its wild type. Thus, this transcript/protein discordance is possibly caused by post-transcriptional regulation (via unknown factors) in an ABA-independent way.

Recently, we found that EMB564 is associated with maize seed vigor (Wu et al., 2011) and is highly thermal stable (Wang et al., 2012). By immunoelctron microscopy, we showed that EMB564 locates preferentially in the nucleus of maize embryonic cells (Wu et al., 2013a). Likewise, Amara et al. (2012) demonstrated that EMB564-green fluorescent protein fusions are expressed in the cytosol and nucleus in the agroinfiltrated leaves of *Nicotiana bentamiana*. Based on bioinformatics analysis, we proposed that EMB564 may function within the nucleus by binding DNA (Wu et al., 2013b).

In addition, we also noticed that some spots are not consistent in abundance between the biological replicates. This inconsistence results mainly from inherit drawbacks of 2-DE (e.g., poor reproducibility between gels) and from minor changes in manipulation during protein extraction among independent biological experiments. However, only those reproducibly, differentially accumulated proteins between *vp5* and *Vp5* are subjected to MS/MS analysis.

### LEA proteins and sHSPs displayed differential accumulations in ABA-deficient embryos

The most interesting finding in this study is that most LEA proteins and sHSPs displayed differential accumulations in ABA-deficient *vp5* embryos: six out of eight identified LEA proteins decreased while nine identified sHSPs increased in abundance, compared to *Vp5*.

LEA proteins are characterized by high hydrophilicity and accumulate to high levels during the last stage of seed maturation (Dure et al., 1989; Espelund et al., 1992). Although the roles of LEA proteins remain speculative, there is evidence supporting their participation in acclimation and/or in the adaptive response to dehydration, low temperature, salinity, or exogenous ABA treatment stress (Battaglia et al., 2008). sHSPs are produced in seeds during maturation and under various stress conditions. The synthesis of sHSPs during seed maturation indicates their probable role in cell component protection mechanisms. Mutants sensitive to desiccation contain smaller amounts of sHSPs during maturation (Wehmeyer and Vierling, 2000).

We tried to explain the possible causes of the observed differential accumulation of six identified LEA proteins and nine identified sHSPs between *vp5* and *Vp5* seeds. Firstly, the threshold content of ABA inducing the accumulation of LEA proteins and sHSPs may differ: some LEA proteins may require higher ABA content than sHSPs during seed maturation. ABA-deficiency in *vp5* embryos greatly inhibited the accumulation of most LEA proteins, but promoted the accumulation of sHSPs. In another words, compared to developmentally specific accumulation of LEA proteins, sHSPs seemed to be less strictly ABA-dependent. In the ABA-deficient *vp5* embryos, EMB564 accumulates to a similar level as its wild type, implying an ABA-independent accumulation of EMB564 in maize embryos. This deduction will be examined by measuring the ABA content and the time-course of accumulation of LEA proteins and sHSPs during *vp5* seed development in future. Previously, differential regulation of ABA-induced 23–25 kDa proteins in embryo and vegetative tissues of maize *vp* mutants was reported (Pla et al., 1989). In a recent study, we identified several proteins in maize *Vp5* and *vp5* seedlings whose syntheses are ABA-independent, such as ADP-dependent malic enzyme and fructose-bisphosphate aldolase (Hu et al., 2012). Maize seeds undergo dehydration process during maturation. ABA mutant *vp5* seeds contain low ABA content and lack obvious dehydration process; therefore, the accumulation of strict ABA-dependent protein (e.g., LEA proteins in this study) may reduce, and not strict ABA-dependent proteins (e.g., sHSPs in this study) may increase to enhance seed dehydration/drought tolerance.

We checked whether there was any difference in the promoter region, especially regarding ABRE and DRE, between differentially accumulated proteins identified here (Tables 5, 6). Most LEA genes are sensitive to ABA because of the presence of ABRE, i.e., regulatory elements in promoter regions, which contain the ACGT sequence called cassette G. Sensitivity to ABA also depends on the presence of MYC elements that include the sequence CACCTG, and MYB elements that include the TAACTG motive (Kalemba and Pukacka, 2007). Dehydration responsive element, DRE, is 9-bp consensus sequence, TACCGACAT, was first identified in the promoter of Arabidopsis rd29A/lti78 and shown to be essential for drought induction in the absence of ABA (Yamaguchi-Shinozaki and Shinozaki, 1994).

**Table 5:** **Cis-acting elements of LEA and sHSPs proteins analyzed by Plantcare**.

**Spot**	**Accession**	**Protein**	**ABRE (Plantcare)**	**Other (Plantcare)**
1	P46517	EMB564	AGTTCGTGGC; GCCACGCACA; CGCACGCGTC; CCGCGTCGGC	
27	K7VM99	Group 3 LEA protein	CCCACGTGTC; CGTACGTGTC; GTCACGTACGT; TACGTG; CACGTG	CAACTG (MBS: MYB binding site involved in drought-inducibility)
2	P24632	HSP 17.8	CACGTG; ACGTGGC; CCAACGTGGC	
4	B6TLK8	HSP 17.4	NO	
5	B6TDB5	HSP 17.4	CCGCGTAGGC; CCGCGTCGGC;CACGTG	TGGCCGAC(C-repeat/DRE)
7	Q08275	HSP 17.0	TACGTG; CACGTG	
8	B6TG53	HSP 22.0	No	CGGGAAGCTTCCAG(HSE); AAAAAATTTA(HSE); CAACTG(MBS: MYB binding site involved in drought-inducibility)
9	B6TXB5	HSP 22.0	TACGTG	CAACTG(MBS: MYB binding site involved in drought-inducibility)
24	B4F976	HSP 17.4	No	CAACTG(MBS: MYB binding site involved in drought-inducibility)
13,14	B6UH67	LEA D34	No	No
22	B6UH99	LEA 14-A	CCAACGTGTC	
29	Q42376	Group 3 LEA protein	GCTACGTGGC; TACGTG; ACGTGGC; CGTTCGTGCA	

**Table 6:** **Cis-acting elements of LEA and sHSPs proteins analyzed by Place**.

**Spot**	**Accession**	**Protein**	**ABRE (Place)**	**DRE (Place)**
1	P46517	EMB564	CACGCGT; CACGCGC	GCCGAC(DRECRTCOREAT)
27	K7VM99	Group 3 LEA protein	ACGTG;TACGTGTC;CACGCGT	ACCGAGA(DRE1COREZMRAB17); ACCGAC(DRE2COREZMRAB17); ACCGAC(DRECRTCOREAT)
2	P24632	HSP 17.8	ACGTG	ACCGAGA(DRE1COREZMRAB17)
4	B6TLK8	HSP 17.4	CACGCGGT	GCCGAC(DRECRTCOREAT)
5	B6TDB5	HSP 17.4	ACGTG	GCCGAC(DRECRTCOREAT)
7	Q08275	HSP 17.0	ACGTG;CACGTGT	ACCGAGA(DRE1COREZMRAB17); ACCGAC(DRE2COREZMRAB17); ACCGAC(DRECRTCOREAT)
8	B6TG53	HSP 22.0	ACGTG	GCCGAC(DRECRTCOREAT)
9	B6TXB5	HSP 22.0	ACGTG;TACGTGTC	ACCGAGA((DRE1COREZMRAB17); ACCGAC(DRE2COREZMRAB17); ACCGAC(DRECRTCOREAT)
24	B4F976	HSP 17.4	NO	NO
13,14	B6UH67	LEA D34	NO	NO
22	B6UH99	LEA 14-A	ACGTG; AACGTGT	GCCGAC(DRECRTCOREAT)
29	Q42376	Group 3 LEA protein	TACGTGGC; ACGTG; ACGTGGCG; ACGTGGCC; AACGTGG	ACCGAC(DRE2COREZMRAB17); ACCGAC(DRECRTCOREAT)

In addition, the accumulation pattern of group 3 LEA protein (spot 27, K7VM99) in *vp5* embryo is different with other five identified LEA proteins. The group 3 LEA proteins are characterized by a repeating motif of 11 amino acids (TAQAAKEKAGE) (Dure et al., 1989), which are quite diverse in sequence structure compared with other LEA groups (Battaglia et al., 2008). In maize *vp5* seeds, group 3 LEA protein accumulation is dependent upon ABA (Thomann et al., 1992), whereas corresponding mRNA has no response to exogenous ABA (White and Rivin, 2000). In maize leaves, *group 3 LEA gene* can be induced by ABA (Liu et al., 2013). Cis-acting elements analysis showed that group 3 LEA protein (K7VM99) shares more identical ABRE and/or DRE with sHSPs (e.g., HSP 17.0) than other LEA proteins (Tables 5, 6). This may explain why group 3 LEA protein (K7VM99) differentially accumulates in *vp5* embryos, like sHSPs but unlike other LEA proteins.

In conclusion, comparative gel-based proteomics revealed significant proteome difference between *vp5* and *Vp5* seeds. Most notably, LEA proteins and sHSPs displayed a differential accumulation pattern in ABA-deficient *vp5* embryos. The data derived from the present study is highly applicable to other crops. The characterization of proteome difference between *vp5* and *Vp5* seeds is necessary for dissection of ABA-mediated maize response in the studies involved *vp5* mutants. The data derived from this study provides insight into ABA-dependent proteins and ABA-mediated response during seed maturation in maize.

### Conflict of interest statement

The authors declare that the research was conducted in the absence of any commercial or financial relationships that could be construed as a potential conflict of interest.
