# Supplementary material for: Proteomic analysis reveals differential accumulation of small heat shock proteins and late embryogenesis abundant proteins between ABA-deficient mutant vp5 seeds and wild-type Vp5 seeds in maize
Source: Front Plant Sci. 2015 Jan 20;5:801. doi: 10.3389/fpls.2014.00801 (PMC4299431; doi:10.3389/fpls.2014.00801)
Supplement: Supplementary file 1 [file Presentation1.PPT]

## Slide 1
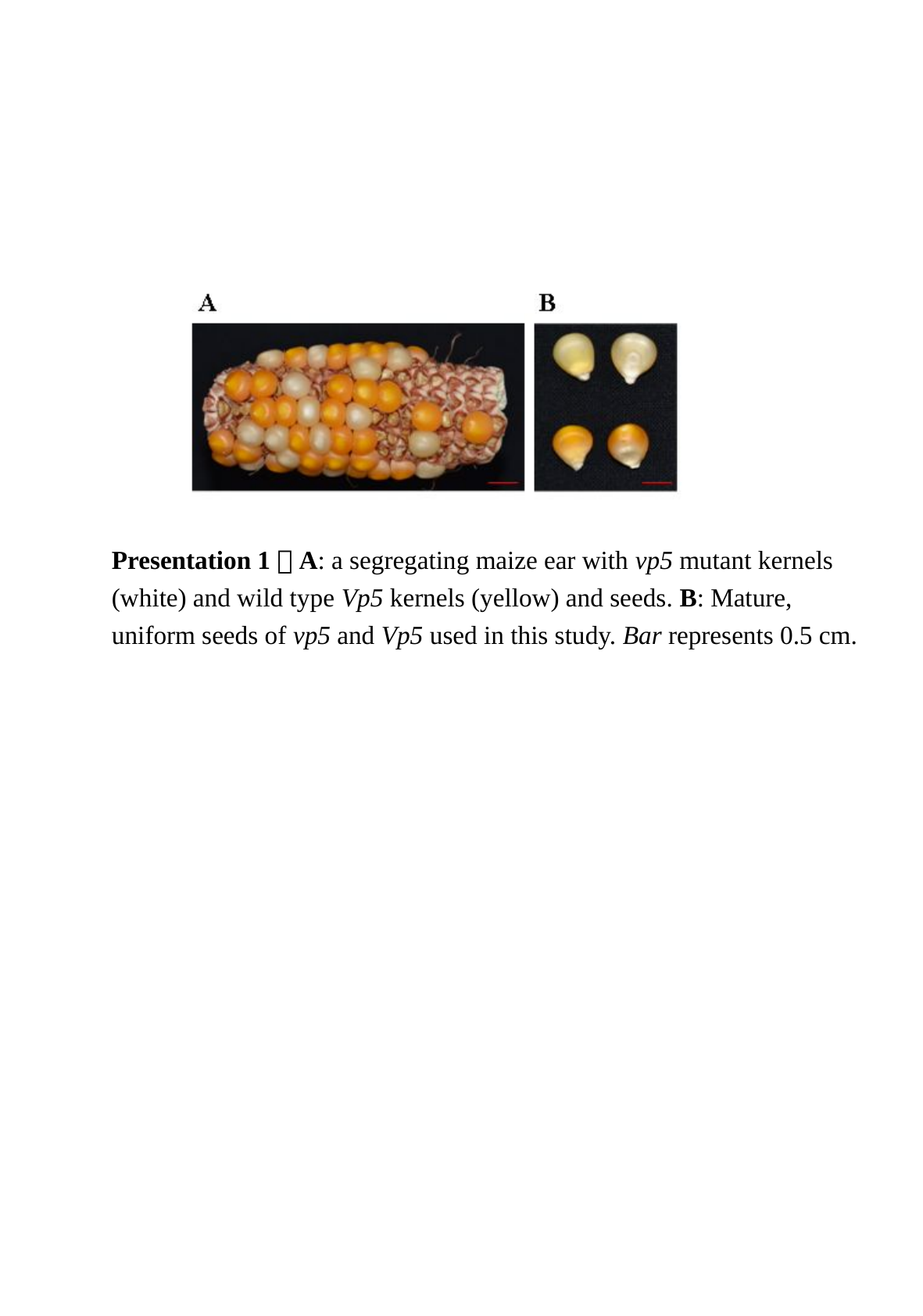

Presentation 1︱A: a segregating maize ear with vp5 mutant kernels (white) and wild type Vp5 kernels (yellow) and seeds. B: Mature, uniform seeds of vp5 and Vp5 used in this study. Bar represents 0.5 cm.
