# Supplementary material for: Proteomic analysis reveals differential accumulation of small heat shock proteins and late embryogenesis abundant proteins between ABA-deficient mutant vp5 seeds and wild-type Vp5 seeds in maize
Source: Front Plant Sci. 2015 Jan 20;5:801. doi: 10.3389/fpls.2014.00801 (PMC4299431; doi:10.3389/fpls.2014.00801)
Supplement: Supplementary file 2 [file Presentation2.PPT]

## Slide 1
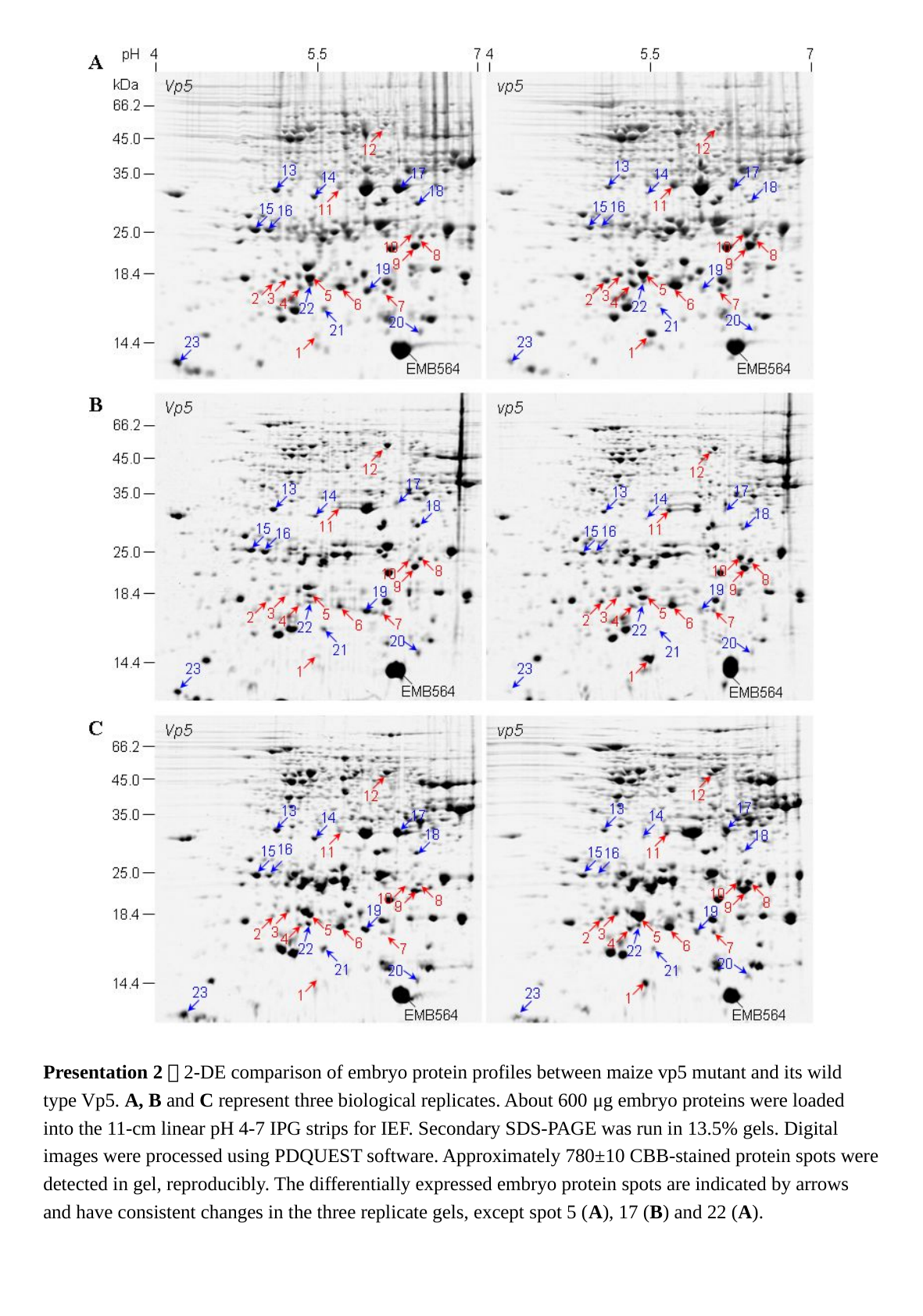

Presentation 2︱2-DE comparison of embryo protein profiles between maize vp5 mutant and its wild type Vp5. A, B and C represent three biological replicates. About 600 μg embryo proteins were loaded into the 11-cm linear pH 4-7 IPG strips for IEF. Secondary SDS-PAGE was run in 13.5% gels. Digital images were processed using PDQUEST software. Approximately 780±10 CBB-stained protein spots were detected in gel, reproducibly. The differentially expressed embryo protein spots are indicated by arrows and have consistent changes in the three replicate gels, except spot 5 (A), 17 (B) and 22 (A).
