# Supplementary material for: Proteomic analysis reveals differential accumulation of small heat shock proteins and late embryogenesis abundant proteins between ABA-deficient mutant vp5 seeds and wild-type Vp5 seeds in maize
Source: Front Plant Sci. 2015 Jan 20;5:801. doi: 10.3389/fpls.2014.00801 (PMC4299431; doi:10.3389/fpls.2014.00801)
Supplement: Supplementary file 3 [file Presentation3.PPT]

## Slide 1
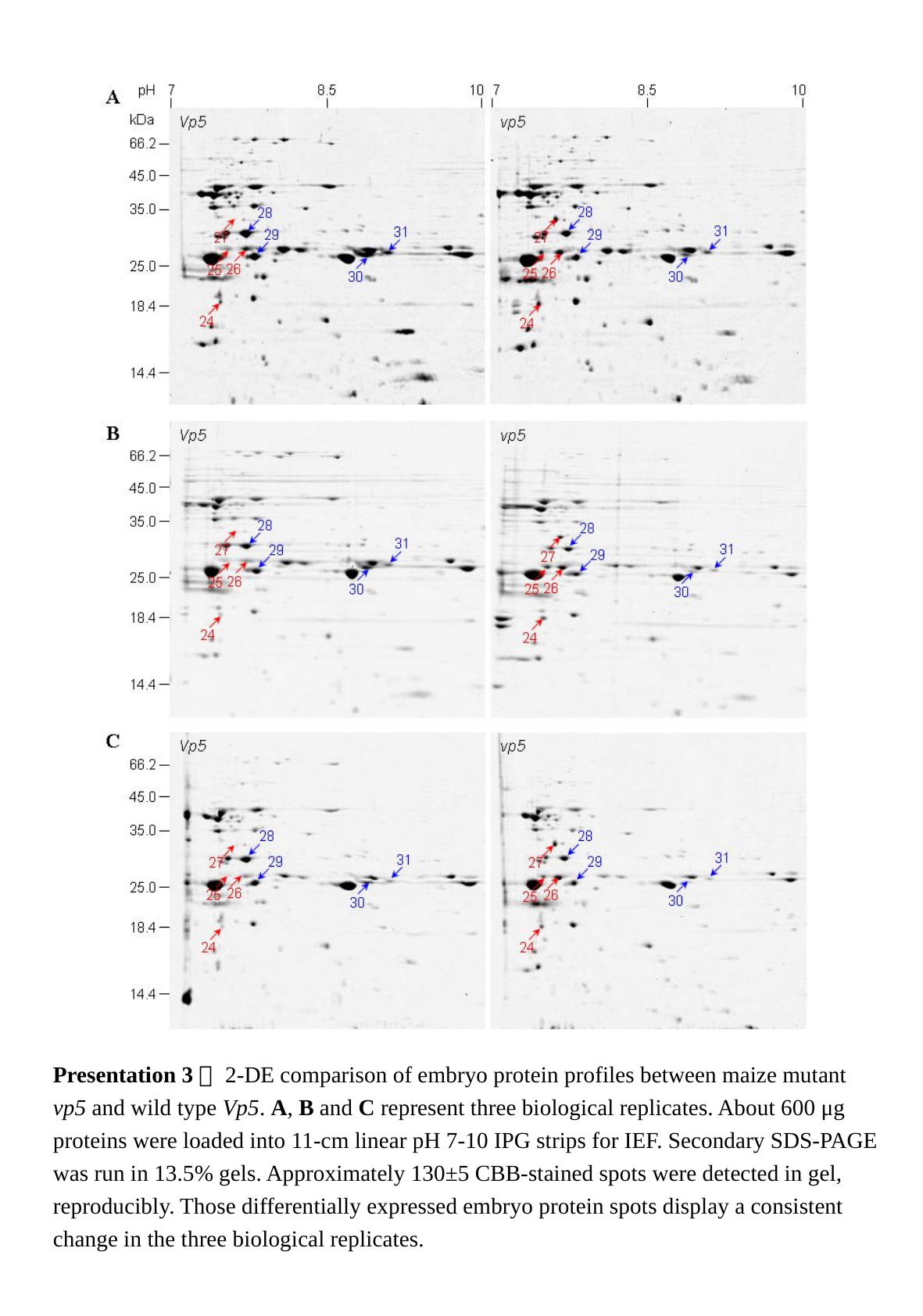

Presentation 3︱ 2-DE comparison of embryo protein profiles between maize mutant vp5 and wild type Vp5. A, B and C represent three biological replicates. About 600 μg proteins were loaded into 11-cm linear pH 7-10 IPG strips for IEF. Secondary SDS-PAGE was run in 13.5% gels. Approximately 130±5 CBB-stained spots were detected in gel, reproducibly. Those differentially expressed embryo protein spots display a consistent change in the three biological replicates.
