# Supplementary material for: Proteomic analysis reveals differential accumulation of small heat shock proteins and late embryogenesis abundant proteins between ABA-deficient mutant vp5 seeds and wild-type Vp5 seeds in maize
Source: Front Plant Sci. 2015 Jan 20;5:801. doi: 10.3389/fpls.2014.00801 (PMC4299431; doi:10.3389/fpls.2014.00801)
Supplement: Supplementary file 4 [file Presentation4.PPT]

## Slide 1
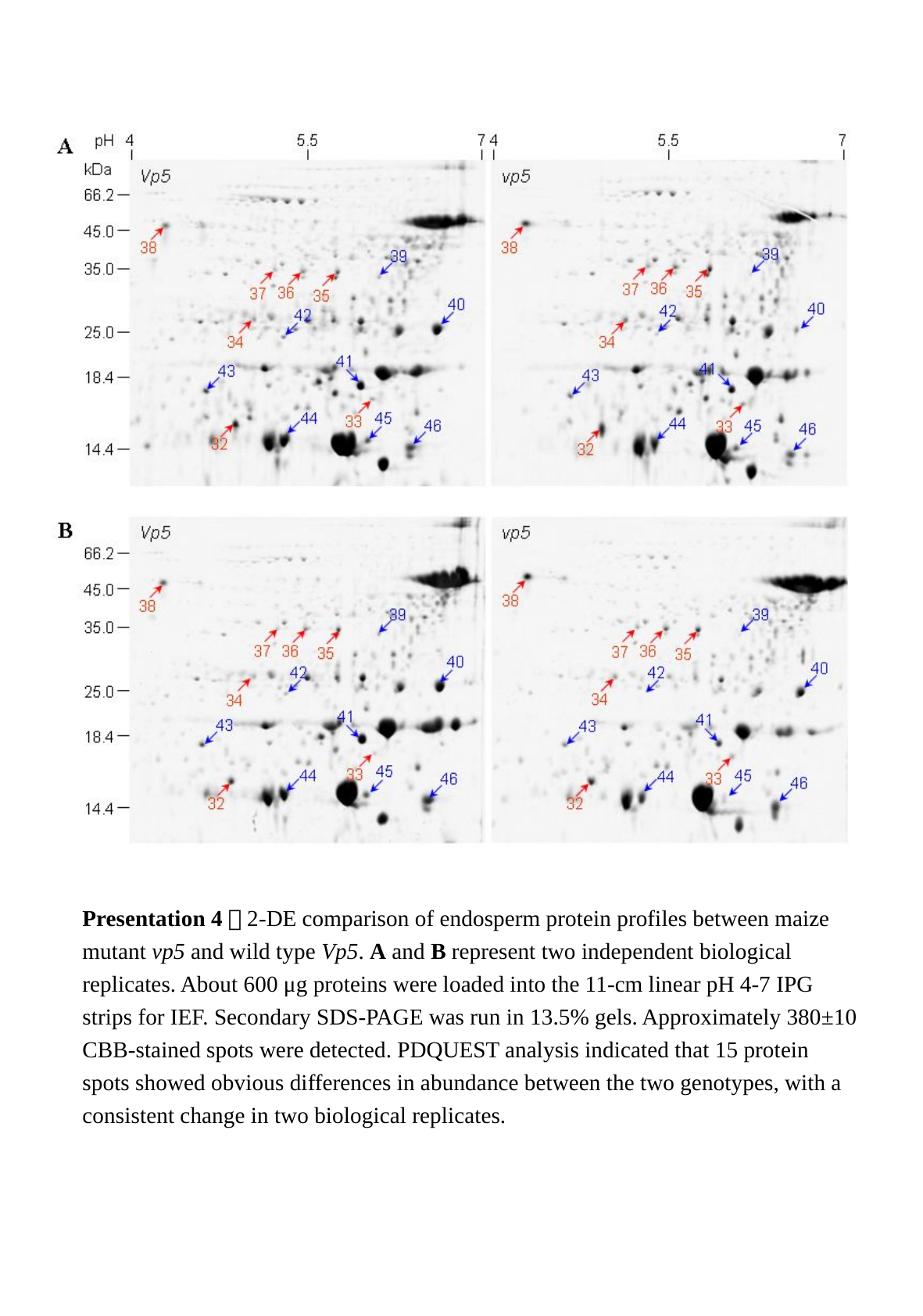

Presentation 4︱2-DE comparison of endosperm protein profiles between maize mutant vp5 and wild type Vp5. A and B represent two independent biological replicates. About 600 μg proteins were loaded into the 11-cm linear pH 4-7 IPG strips for IEF. Secondary SDS-PAGE was run in 13.5% gels. Approximately 380±10 CBB-stained spots were detected. PDQUEST analysis indicated that 15 protein spots showed obvious differences in abundance between the two genotypes, with a consistent change in two biological replicates.
